# Structural and functional characterization of recombinant human growth hormone isolated from transgenic pig milk

**DOI:** 10.1371/journal.pone.0236788

**Published:** 2020-07-31

**Authors:** So-Young Lee, Joo-Hee Han, Eun-Kyeong Lee, Young Kyu Kim, Seo-Ah Hwang, Sung-Hyun Lee, Maria Kim, Gye Yoon Cho, Jae-Ha Hwang, Su-Jin Kim, Jae-Gyu Yoo, Seong-Keun Cho, Kyung-Ju Lee, Weon-Ki Cho

**Affiliations:** 1 CHO-A Biotechnology Research Institute, CHO-A Pharmaceutical Company, Yeoju-si, Gyeonggi-do, Korea; 2 Department of Animal Science, College of Natural Resources and Life Science, Pusan National University, Miryang-si, Gyeongsangnam-do, Korea; 3 Animal Diseases and Biosecurity Team, National Institute of Animal Science, Rural Development Administration, Wanju-gun, Jeollabuk-do, Korea; Friedrich-Loeffler-Institute, GERMANY

## Abstract

This study aimed to establish and reproduce transgenic pigs expressing human growth hormone (hGH) in their milk. We also aimed to purify hGH from the milk, to characterize the purified protein, and to assess the potential of our model for mass production of therapeutic proteins using transgenic techniques. Using ~15.5 L transgenic pig milk, we obtained proteins with ≥ 99% purity after three pre-treatments and five column chromatography steps. To confirm the biosimilarity of our milk-derived purified recombinant hGH (CGH942) with commercially available somatropin (Genotropin), we performed spectroscopy, structural, and biological analyses. We observed no difference between the purified protein and Genotropin samples. Furthermore, rat models were used to assess growth promotion potential. Our results indicate that CGH942 promotes growth, by increasing bone development and body weight. Toxicity assessments revealed no abnormal findings after 4 weeks of continuous administration and 2 weeks of recovery. The no-observed-adverse-effect level for both males and females was determined to be 0.6 mg/kg/day. Thus, no toxicological differences were observed between commercially available somatropin and CGH942 obtained from transgenic pig milk. In conclusion, we describe a transgenic technique using pigs, providing a new platform to produce human therapeutic proteins.

## Introduction

Human growth hormone (hGH), synthesized in the pituitary gland, is composed of 191 amino acids. This hormone plays a vital role in growth and development, contributing to bone development and muscle gain [1–3]. Since endogenous hGH is a non-glycosylated protein, early attempts to induce its overexpression have been performed in *Escherichia coli* [4]. However, recombinant hGH (rhGH) in this system was expressed in the periplasmic space [5] or in the form of insoluble inclusion bodies, together with other eukaryotic proteins [6, 7], making it necessary to utilize onerous solubilization and purification processes. Until now, multiple studies have attempted to induce the expression of soluble rhGH using different host systems, including *Bacillus subtilis* [8], mammalian cells [9], baculovirus systems [10], and yeast cultures [11].

In the early 1990s, an attempt was made to generate a transgenic animal model that could express various human proteins [12]. The first transgenic animal model was successfully produced via microinjection of genetically modified DNA into pronucleus of mouse zygote [13]. However, the efficiency of transgenic animals’ production from a surrogate mother using microinjection of modified DNA into zygote was extremely low. Consequently, various surgical procedures, numerous experimental animals, and expert-level techniques were required to obtain transgenic animals [12].

In 1997, a cloned sheep was produced by nuclear transfer (NT) of a somatic mammary gland cell into an oocyte [14]. Although this method used somatic cells, it allowed the potential modification of donor cells via cellular transfection and selection procedures, and therefore the generation of locus-specific transgenic animals via nuclear transfer of these donor cells. This method was cost-effective and straightforward for producing of transgenic animals [12].

Previous studies of recombinant proteins produced using transgenic animals targeted mostly plasma proteins such as albumin [15], granulocyte-colony stimulating factor [16], coagulation factors [17], and erythropoietin [18, 19]. To conveniently separate and purify transgenic animals-derived recombinant proteins, tissue-specific expression was induced using mainly beta-casein or whey acidic protein (WAP) promoters in secretory organs such as the mammary glands.

Multiple studies aimed to develop transgenic animal models expressing rhGH in milk. These models included rhGH expression in goats using the goat beta-casein promoter [20], transgenic cows using the cow beta-casein promoter [21], and transgenic rabbits using the rat whey acidic protein promoter [22]. However, no follow-up studies have been reported. In 2006, GTC Biotherapeutics produced human anti-thrombin secreted from transgenic goats as a biomedical product and obtained approval for production and commercialization in Europe. This product was approved by the FDA 3 years later under the brand name ATryn and became commercially available for patients. This example highlighted the importance of transgenic animals as bioreactors, and their potential to produce therapeutic proteins.

The current study was conducted to assess the feasibility of transgenic pigs as bioreactors for producing of therapeutic proteins. We demonstrated the utility of this model by confirming the efficacy and safety of rhGH produced using this system.

## Materials and methods

### Ethics statement

All animal procedures were conducted in accordance with the Animal Protection Act of Korea. Each experiment was approved by the CHO-A Biotechnology Research Institute ethical committee (Choa-Bio-00-002) and the Biotoxtech Institutional Animal Care and Use Committee (170618, 170433).

#### Pigs

Landrace pigs were used for all experiments. Food was provided twice per day, with *ad libitum* access to water. The environment was maintained at 28°C. All surgeries were performed under Zoletil (Vibac, France) anesthesia and maximum care was taken to minimize animal suffering.

#### Rats

Sprague-Dawley wild type and hypophysectomized rats (SLC Inc., Japan) were purchased from Central Lab. Animal Inc. (Korea). Two-to-three rats were housed in polycarbonate cages under 12-h light/12-h dark cycles with *ad libitum* access to sterile food and water. Each rat was single-housed in the conditions described above during the experimental period.

### Plasmid construction

The pPBC vector and *WPRE* (woodchuck hepatitis virus post-transcriptional regulatory element) gene construct were prepared as per a previously described method [23]. This vector contained the pig beta-casein promoter, 5'-untranslated region (UTR), 3'-UTR, polyadenylation signal, ribosome binding sequence, intron, a selective marker, and a neomycin cassette. rhGH was cloned from human cDNA, fused with *WPRE* using the *EcoR*V site, and inserted into the pPBC vector. The pPBC-hGH-W vector was constructed by inserting *hGH-WPRE* into the *Xho*I site of the pPBC vector. All constructs were confirmed by sequencing.

### Establishment of stably transfected cells

The establishment of stable *pPBC-hGH-W*-transfected cells was performed as per a previously described method [24]. Prior to transfection, *pPBC-hGH-W* was linearized with the *Sal*I restriction endonuclease (New England Biolabs, Ipswich, MA, USA) and separated on a 1% agarose gel. The plasmid was then purified with a gel extraction kit (QIAGEN, Hilden, Germany). For electroporation, 5 × 10^6^ porcine fetal fibroblasts (PFF) [25] were collected at 70% confluence, mixed with 10 μg DNA, transferred into a 100-μL Neon tip (Invitrogen, CA, USA), and subjected to one 1.55 kV pulse for 20 ms delivered by a microporator (Digital Bio, Seoul, Korea). Electroporated cells were cultured in Dulbecco’s modified Eagle medium (DMEM) (Hyclone, Logan, UT, USA) containing 10% fetal bovine serum (FBS) (Tissue Culture Biologicals, Tulare, CA, USA), 100 U/mL penicillin, and 100 μg/mL streptomycin (Hyclone, Logan, UT, USA) at 38.5°C in 5% CO_2_ and humidified air. After selection with 700 μg/mL-800 μg/mL G418 (Gibco, Waltham, MA, USA) for 14 days, several transgenic single cell colonies were isolated and expanded for somatic cell nuclear transfer (SCNT). A portion of the cells was analyzed by PCR to identify transfected cells after the selection of single colonies. The remaining cells were expanded by passaging until sufficient cells were obtained for cryopreservation. Genomic DNA was extracted by enzymatic lysis using proteinase K (QIAGEN, Hilden, Germany), and was then used as a PCR template. PCR analysis was performed using G-Tag polymerase (Labopass, Seoul, Korea) and the primer pairs described in Table 1. The PCR conditions were as follows: 94°C for 2 min, followed by 30 cycles of 94°C for 30 s, 56°C for 30 s, and 72°C for 1 min. Each PCR sample was resolved on 1% agarose gels by electrophoresis. The PCR analysis was repeated three times, and only cells with similar results were considered as transgenic cell lines.

**Table 1:** Primer pairs for PCR analysis.

Primer Name	Sequence (5′→3′)	GC (%)	Tm (ºC)	Product Length (bp)
hGH-F	TGCAGTTCCTCAGGAGTGTCT	52.4	57.8	414
WPRE-R	GCATTAAAGCAGCGTATCCAC	47.6	55.9	
PBC5ʹdown-F	GGATAAGGCTGTTAGTGGAAA	42.9	53.9	760
hGH-R	GTCATCGTTGTGTGAGTTTGT	42.9	53.9	
WPRE-F	TGGCTAAATGGTGCTGTATAA	38.1	51.9	462
PBC3′up-R	TAAGAGTCCTCACCACTCCTC	52.4	57.8	

### Oocyte collection and *in vitro* maturation

Pre-pubertal gilt ovaries were collected from a slaughterhouse (Woo Jin Industries Co., Ltd., Gyunggi-do, Korea), stored at 28°C, and transported to the laboratory within 1 h for further processing. Oocytes were collected as per a previously described method [26] and matured via culture with North Carolina State University-23 (NCSU-23) [27] medium for 42 h–44 h.

### SCNT and *in vitro* development

SCNT in mature oocytes was performed as per a method described previously [25]. After electrofusion, *in vitro* development was performed using Porcine Zygote Medium 5 (IFP, Yamagata, Japan) containing 0.4% bovine serum albumin (Sigma, St. Louis, MO, USA). After culturing for 1–2 days, cleaved embryos were selected and transferred into surrogate mothers.

### Preparation of surrogate mothers, transgenic cloned embryo transfer, and diagnosis of pregnancy

Surrogate mothers were 7–8-month-old gilts with a normal estrus cycle, as described in a previous study [28]. 1–4-cell stage transgenic cloned embryos were transferred into the oviduct of surrogate mothers. Pregnancy was assessed by ultrasound 25–30 days after transfer.

### Fluorescence in situ hybridization (FISH)

Nick translation (DIG-Nick Translation Mix, Roche Diagnostics) was used to obtain labeled probes for FISH [29]. Blood was collected from the jugular vein of rhGH transgenic pigs with a syringe pre-loaded with heparin (Sigma, Aldrich, St. Louis, MO, USA). Then, blood (0.5 mL) was mixed into the culture solution and incubated at 37°C and 5% CO_2_ for 72 h. The cultures were grown in RPMI 1640 (Hyclone, Logan, UT, USA) with 10% FBS, 1% penicillin-streptomycin (Sigma, St. Louis, MO, USA) and 2% lectin (Sigma, St. Louis, MO, USA). One hour before the end of incubation, cells were treated with 0.5 μg/ml colcemid (Demecolcine, Sigma) to arrest mitosis in the metaphase stage. 10 mL of 0.06 M KCl (Sigma, Aldrich, St. Louis, MO, USA) was added and incubated for 15 min at room temperature. Then, cells were treated 2–3 times with a 3:1 methanol (Merck KGaA, Darmstadt, Germany) and acetic acid (Junsei, Tokyo, Japan) mixture to remove suspended solids and fix the cells. After centrifuging at 1200 × *g* for 5 min, the cell pellets were resuspended, mounted onto a glass slide, and air-dried. Samples were then washed and dehydrated with ethanol (Merck KGaA, Darmstadt, Germany) after treatment RNase (Bioneer, Daejeon, Korea). Then, the samples were incubated at 85°C for 10 min in hybridization solution Then, the samples were incubated at 85°C for 10 min in hybridization solution [0.7 M NaCl, 0.1 M Tris pH 8.0, 0.1% SDS, and 10 mM EDTA (all from Sigma Aldrich, St. Louis, MO, USA)] and bonded at 38°C for 12 h. After conjugation, the slides were washed with PBS (AMRESCO, Solon, OH, USA) pH 8.0 for 5 min at 72°C, and then treated with anti-digoxigenin-fluorescein (Sigma Aldrich, St. Louis, MO, USA) and covered with glass. The reaction was carried out at 38°C for 30 min. Slides were then washed with PBS and dried in the dark. Nuclei were stained with propidium iodide (Sigma Aldrich, St. Louis, MO, USA) solution. Finally, the samples were covered with coverslip and dried in the dark. Fluorescent conjugation was observed using a fluorescence microscope (Olympus, Tokyo, Japan) with a 523-nm wavelength filter.

### Verification of transgenic piglets

The presence of transgenes in produced piglets was confirmed by PCR (Takara, Shiga, Japan), using genomic DNA extracted from the umbilical cord. Genomic DNA was extracted with an Exgene Tissue SV Kit (GeneAll, Seoul, Korea). Some primers used (hGH-WPRE forward and reverse, PBC 5′ down forward, and hGH reverse) are listed in Table 1. The PBC 3′up forward (5′-ATGCCTTTGTATCATGCTATTGCT-3′) and PBC 3′up reverse primers (5′-ATGGAATTTGCCTTTATTTTAGGCT-3′) were used to amplify the predicted 657-bp product, which was then confirmed using 1% agarose gel electrophoresis. The amplification conditions were as follows: 5 min at 95°C, 32 cycles at 94°C for 30 s, 56°C for 30 s, and 72°C for 1 min, with a final extension for 5 min at 72°C. The PCR product was visualized with an Illuminator UV (Spectroline, Westbury, NY, USA).

### Collection of transgenic pig milk

Milk was collected from day 1 to day 45 of the lactation period. Prior to milk collection, 2 mL of oxytocin (Bayer, Ansan, Korea) was injected into the ear vein of the sow to induce milk ejection. The milk was collected in a 50-mL tube (Falcon, Mexico City, Mexico) twice daily. Then, 1 mL of the collected milk was transferred into an E-tube for rhGH quantification. The remaining milk was stored in a –70°C deep freezer (Shinshin Biobase, Korea). After quantification, only the milk with rhGH > 50 μg/mL was collected from the respective transgenic sows.

### Pretreatment of transgenic sow milk (sample preparation)

The transgenic milk samples were diluted with a 2-fold volume of EDTA (Sigma, St. Louis, MO, USA) at a final concentration of 20 mM to extract rhGH within casein micelles. The treated milk was centrifuged at 11,000 × *g* for 1 h at 4°C. Defatted milk samples were acidified by slowly adding 50% acetic acid (Merck Millipore, Billerica, MA, USA) with constant stirring until the milk reached pH 4.25, which precipitated casein. The samples were then centrifuged at 11,000 × *g* for 1 h at 4°C. The whey samples were neutralized using 2 M Tris (AMRESCO, Solon, OH, USA) and filtered through a 0.2-μm hollow fiber membrane microfilter (GE Healthcare Life Sciences, Little Chalfont, UK). Samples containing rhGH were clarified by tangential flow filtration with a nominal 300-kDa pore size hollow fiber membrane ultrafilter (GE Healthcare Life Sciences, Little Chalfont, UK). The 300-kDa filter permeate was concentrated and diafiltered through a nominal 5-kDa cut-off size hollow fiber membrane ultrafilter (GE Healthcare Life Sciences, Little Chalfont, UK). The supernatant from each step was analyzed by SDS-PAGE using 13.5% (w/v) gel under reducing conditions. Proteins were stained with Coomassie brilliant blue R-250 (AMRESCO, Solon, OH, USA).

### Determination of rhGH concentration

Recombinant hGH was indirectly quantified using an hGH ELISA kit (Quantikine ELISA human growth hormone; R&D Systems, Minneapolis, MN, USA) following the manufacturer’s protocol. Assay readouts were measured spectrophotometrically at 450 nm using a microplate spectrophotometer (Model 680, Bio-Rad, Hercules, CA, USA). The samples were diluted 10^−5^–10^−7^-fold with distilled water.

Samples were prepared for Western blot analysis as follows: raw milk was diluted 10–100 fold with distilled water and centrifuged. The supernatant was collected and loaded onto 12% or 13.5% polyacrylamide gels and separated by electrophoresis. Proteins were transferred to PVDF membranes (Immobilon-P, Millipore, Burlington, MA, USA) using an ATTO Blotting System (ATTO, Tokyo, Japan). Then, the PVDF membrane was incubated in Tris-buffered saline (TBS, 20 mM Tris, 150 mM NaCl, pH 7.6) containing 5% skim milk and 0.1% Tween-20 (AMRESCO, Solon, OH, USA) for 1 h at room temperature. Next, the membrane was incubated with mouse anti-hGH primary antibody (1:3000–1:10000, R&D systems, Minneapolis, MN, USA), followed by anti-mouse horseradish peroxidase-conjugated secondary antibody (1:3000–1:5000, Santa Cruz, USA). Protein bands were identified using Luminol reagent (Immuno Cruz, Santa Cruz Biotechnology, Dallas, TX, USA).

### Purification of rhGH by 5-step column chromatography

After pretreatment, milk samples were mixed and centrifuged at 4°C and 7000 × *g* for 15 min to remove the precipitated casein. The pH was set to 4.8 and conductivity was set to 3.0 mS/cm. The mixed sample was then applied to a CM Sepharose column (CV 3.5 L, GE Healthcare). After the column was washed with 30 mM NaCl in 20 mM sodium acetate (pH 4.8), the protein fraction containing rhGH was eluted with 90 mM NaCl in 20 mM sodium acetate (pH 4.8). The active fraction was equilibrated with a buffer for Nickel Sepharose, the secondary column, using a UF membrane (0.5 m^2^, 5 kDa MWCO, PES, Millipore). The samples were then applied to a Nickel Sepharose 6FF column (CV 1.5L, GE Healthcare, Uppsala, Sweden), pre-equilibrated with 0.5 M NaCl in 20 mM sodium phosphate (pH 8.3). The column was washed with the same buffer and the active fraction was eluted with 0.5 M NaCl in 20 mM sodium acetate buffer (pH 6.25). The remaining proteins were eluted with 0.5 M NaCl in 20 mM sodium acetate pH 4.8 to regenerate the column. All active fractions were pooled and the buffer was exchanged using a UF membrane in the same manner. The third column used was an ANX Sepharose column (CV 1L, GE Healthcare Uppsala, Sweden), pre-equilibrated with 30 mM NaCl in 20 mM Tris (pH 8.5). The samples were exchanged with the same buffer, and the column was washed with 70 mM NaCl in 20 mM Tris (pH 8.5). Protein fractions containing rhGH were eluted with 130 mM NaCl in 20 mM Tris (pH 8.5). The fourth column used was a Resource phenyl column (CV 0.375L, GE Healthcare Uppsala, Sweden), which was equilibrated with 1 M (NH_4_)_2_SO_4_ in 20 mM Tris (pH 8.2). Washing unbound protein and impurities was performed in 2 steps using 20 mM Tris containing 20% 2-propanol/0.6 M (NH_4_)_2_SO_4_ and 24% 2-propanol/0.52 M (NH_4_)_2_SO_4_. The protein fractions containing rhGH were eluted with 30% 2-propanol/0.42 M (NH_4_)_2_SO_4_ buffer, pooled, and diluted 3-fold with 20 mM Tris to prevent protein damage. After concentration and PBS buffer exchange using UF membranes (as described above), the sample was applied to the last column, a Sephacryl S-100 column (CV 6.8L, GE Healthcare Uppsala, Sweden). Protein elution was performed using two column volumes. The fraction containing rhGH was collected and concentrated by UF membrane filtering. The final purified sample was dissolved in excipient (glycine 2.2 mg/mL, NaH_2_PO_4_ 0.32 mg/mL, Na_2_HPO_4_ 0.31mg/mL, and mannitol 1.8 mg/mL), filtered, and lyophilized.

### Characterization of purified rhGH (CGH942)

To identify the purified CGH942 protein structure, we performed multiple structural analyses. Total mass, N-terminal sequencing, C-terminal sequencing, RP-HPLC, and peptide map analysis were performed by Biosystems, Inc. (Korea). Circular dichroism (CD) spectropolarimetry, UV spectrum analysis, and Fourier transform-infrared (FT-IR) analysis were performed by Korea Basic Science Institute (KBSI, Korea).

### Cell proliferation assay

Nb2-11 cells were cultured in Fischer's medium with 1% FBS, 10% horse serum, 1% penicillin- streptomycin, and 50 μM β-mercaptoethanol (Sigma Aldrich, St. Louis, MO, USA) for 48 h in an incubator at 37°C and 5% CO_2_. The cultured cells were centrifuged to remove the supernatant and washed twice with 5 mL PBS. Then, 5 × 10^4^ cells per well were added to a 96-well plate in Fisher's medium without FBS, and incubated at 37°C and 5% CO_2_ for 24 h. The cells were treated with Genotropin INJ 16IU (Pfizer, Sweden) and CGH942 at concentrations of 0.01 ng/mL to 1000 ng/mL and incubated for another 48 h. Then, 10 μL of the MTT solution was added to each well and allowed to react for 4 h. Absorbance was measured at a wavelength of 415 nm.

### Growth enhancement efficacy test

Rats were left untouched (wild type control group) or hypophysectomized. Hypophysectomized mice were then separated into two groups: the negative control and experimental groups. CGH942 (0.035 mg/kg, 0.073 mg/kg, and 0.146 mg/kg) and Genotropin (0.146 mg/kg) were only administered to the experimental group. Distilled water containing excipient was administered to the control groups. All treatments were administered once daily for 21 days. The dose was set to 1 mL/kg based on the individual weight measured near the day of administration. During the test, general features such as the eye, skin, hair and behavior were observed once per day. Any animal that died during the experiment was subjected to necropsy. Before injection, rats were weighed. At the end of the experiment, animals were anesthetized with isoflurane and sacrificed by aortic puncture. Tibia extraction was performed post-mortem. 20 mg/kg tetracycline hydrochloride (Sigma Aldrich, St. Louis, MO, USA) was intraperitoneally administered 72 h before tibia extraction. The length of the extracted tibiae was measured using a caliper (BLUEBIRD, China). The extracted tibiae were fixed in 4% paraformaldehyde (Sigma, St. Louis, MO, USA) for 48 h and decalcified in 10% EDTA (Deajung Chemical & Metals Co., Ltd., Siheung, Korea) for 24 h, followed by dehydration in 30% sucrose for 24 h. Dehydrated tibiae were then sliced into 40-μm thick longitudinal sections with a freeze-cutting machine (Leica, Wetzlar, Germany). The tibiae sections were then attached to a glass slide, mounted with Fluoromount (Sigma Aldrich, St. Louis, MO, USA) and overlaid with coverslips. Preparations were observed with a fluorescence microscope (Observer Z1, Carl Zeiss, Oberkochen, Germany). Histologic photographs were acquired (Axiovision V.4.6, Carl Zeiss, Germany). The lengths of the bands produced by tetracycline in the growth plate were measured using Axiovision V.4.6 software (Carl Zeiss, Germany).

### Evaluation of toxicity and toxicokinetics

Toxicity tests were performed as per the CDER guidelines (Center for Drug Evaluation and Research, application, NDA 21–148, NDA 21–075) and as described previously [30]. CGH942 was administered in three dosages (one per group of animals): 0.15 mg/kg/day, 0.3 mg/kg/day, and 0.6 mg/kg/day and Genotropin was administered at 0.6 mg/kg/day. Each group consisted of 10, 10, 15, and 15 animals, respectively. Male/female Sprague-Dawley rats were included in each group and received the drugs subcutaneously once daily for four weeks. The amount of administered solution was calculated as per the animals’ body weights. Drugs were injected into the subcutaneous tissue of the neck. In parallel, wild type female and male rats, received daily subcutaneous injections of PBS during four weeks, as the control group. To evaluate the reversibility of toxicity, 5 males and 5 females were injected with 0.6 mg/kg/day of CGH942 and Genotropin, and then allowed to recover for 2 weeks. Throughout the observation period, we evaluated general signs, body weights and food intakes, and performed ophthalmological examinations and urine tests. After the observation period, we performed hematological examinations, blood biochemical tests, organ weight measurements, and post-mortem macroscopic and histopathological examinations. Blood collection and plasma separation were performed at 0 h, 0.25 h, 0.5 h, 1 h, 3 h, 6 h, 10 h, and 24 h on the first day of administration and after 4 weeks of drug administration. Blood (approximately 0.5 mL) was collected from the jugular vein using a 1-mL syringe treated with heparin sodium (10 IU/mL–20 IU/mL). Plasma was separated by centrifugation at 4°C for 3 min at 14,000 × *g*. The separated plasma was divided into two micro-tubes and kept frozen at –70°C until further use. Plasma CGH942 and Genotropin levels were analyzed using an ELISA kit (AVIVA SYSTEMS BIOLOGY, San Diego, CA, USA). These analyses were carried out by Biotoxtech (Cheongju-si, Korea), and SCAS-BTT Bioanalysis Co., Ltd. (Cheongwon-gun, Korea).

### Statistical analysis

In the toxicity study, the body weight, longitudinal bone growth, and tibia length data were statistically analyzed using SAS (version 9.3, SAS Institute Inc., USA). The wild type control group (G1) and the hypophysectomized negative group (G2) were subjected to the Folded-F assay to test for equivariance. The Student’s *t*-test was performed in the case of equivariance (significance level: 0.05 and 0.01 for one side), and the Aspin-Welch t-test was performed if equivariance was rejected (significance level: 0.05 and 0.01 on both sides). Equal variance between each test group (G3 ~ G5) for the negative control group (G2) was tested using the Bartlett’s test (significance level: 0.05), one-way analysis of variance (ANOVA) (significance level: 0.05), the Dunnett’s t-test (significance level: 0.05 and 0.01 for one side), the Kruskal-Wallis test, and the Steel’s test (significance level: 0.05 and 0.01 for one side).

## Results

### Somatic cell line construction and production of rhGH transgenic pigs

**Fig 1:**
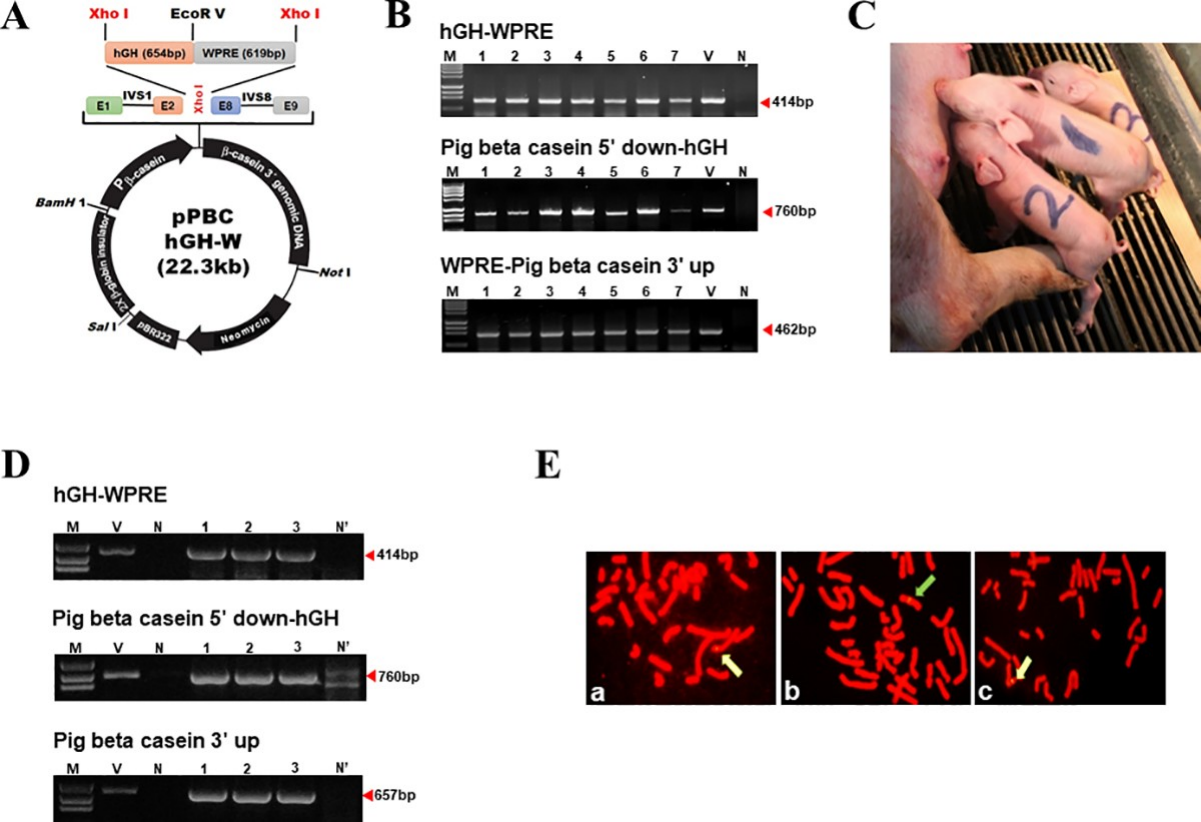
Production of human growth hormone transgenic pigs using SCNT. (A) Schematic of the pPBC-hGH-W vector. (B) Confirmation of transgene integration in transfected cells by PCR. M: size marker; V: pPBC-hGH-W vector as the positive control; N: Wild type PFF cells as the negative control; Lanes 1–7: porcine fetal fibroblasts transfected with pPBC-hGH-W constructs. (C) Identification of transgenic cloned rhGH-piglets. Three cloned transgenic pigs, 2 days after birth. (D) Identification of transgenic piglets by PCR using genomic DNA collected from piglets’ umbilical cords. Lanes 1–3 show the transgene detected in transgenic piglets. (E) Arrows indicate the localization of the rhGH gene in the cloned piglet chromosomes. a; Piglet 1, b; Piglet 2, c; Piglet 3 (from Panel C) 5 days after birth.

Production of rhGH transgenic pigs was performed using a pPBC vector synthesized in our lab [23]. The *hGH* gene cloned from human cDNA was ligated to *WPRE* using an *Eco*RV enzyme site. This ligated product was inserted into the pPBC vector to create the final vector (Fig 1A). The pPBC-hGH-W construct was linearized and randomly inserted into PFF cells isolated from a 30-day-old pig fetus to generate a transgenic cell line. Insertion of the transgenic gene into PFF cells was confirmed by PCR (Fig 1B).

Using the generated transgenic somatic cells, we first compared cleavage and blastocyst production during *in vitro* development between wild type donor cells (non-transfected) and transgenic somatic cells (rhGH transfected) (Table 2). The fusion rate (84.8% vs. 72.7%), cleavage rate (83.6% vs. 48.6%), and blastocyst rate (8.5% vs. 6.4%) were more efficient in the non-transfected group than those in the rhGH transfected group. Then, rhGH insertion was confirmed by PCR using genomic DNA extracted from the umbilical cord of the produced piglets (Fig 1C and 1D). Finally, the insertion of rhGH was confirmed by fluorescence in situ hybridization (Fig 1E).

**Table 2:** *In vitro* development of transgenic pig embryos for SCNT.

Cell type	No. of oocytes injected	No. of	No. of	No. of
		fused (%)	cleavage (%)	blastocysts (%)
Wild type ^a^	223	189 (84.8)	158 (83.6)	16 (8.5)
Transgenic ^b^	495	360 (72.7)	175 (48.6)	23 (6.4)

^a^ Non-transfected

^b^ rhGH transfected

Ten individually-established PFF cell lines were used as donor cells and cleaved embryos (after SCNT) were transferred into 114 surrogate mothers. Overall, 146 piglets were produced, and 69 piglets were confirmed to be transgenic (Table 3). The transgenic piglets were raised until puberty, and artificial insemination (AI) was performed to assess germline transmission and recombinant protein secretion into the milk.

**Table 3:** Transgenic cloned embryos: Transfer result.

Donor cell colonies	Recipient pigs	No. of embryos transferred	Viable offspring (%)	Transgenic offspring (%)	Transgenic/viable offspring -%
hGH-LYY13	6	2,006	15 (0.75)	11 (0.55)	73
hGH-LYY15	12	4,288	6 (0.14)	6 (0.14)	100
hGH-LYY17	36	13,391	44 (0.33)	18 (0.13)	40.9
hGH-LYY21	19	6,555	24 (0.37)	9 (0.14)	37.5
hGH-LYY22	7	3,129	14 (0.45)	0 (0.00)	0
hGH-LYY25	2	552	0 (0.00)	0 (0.00)	0
hGH-LYY27	8	2,819	21 (0.74)	13 (0.46)	61.9
hGH-LYY28	4	1,294	0 (0.00)	0 (0.00)	0
hGH-LYY29	14	3,574	10 (0.28)	9 (0.25)	90
hGH-LYY58	6	1,549	12 (0.77)	3 (0.19)	25
Total	114	39,157	146 (0.37)	69 (0.18)	47.2

In the F0 generation, 16 sows gave birth to 175 piglets, with each sow producing 10.9 piglets on average. In total, there were 79 transgenic piglets, this is a 45.1% transgenic rate. In the F1 generation, there were 46 piglets produced from 5 sows, with each sow producing 9.2 piglets on average. Among them, 25 piglets were confirmed to be transgenic, indicating a 54.3% transgenic rate (Table 4).

**Table 4:** Analyses of transgenic gene transfer in pigs.

Generation	Recipient sows	Offspring	Litter size	Transgenic (%)
F0	16	175	10.9	79 (45.1)
F1	5	46	9.2	25 (54.3)

### Confirmation of rhGH protein expression in transgenic pig milk and pretreatment of transgenic sow milk

We assessed rhGH expression in transgenic pigs’ (F0) milk by Western blotting and ELISA. Although identical vectors were used, there were differences in the rhGH expression levels comparing individual animals generated with the different established cell lines. The rhGH concentration varied between 7.8 μg/mL and 1.7 mg/mL according to the cell line used. Of note, the amount of rhGH expression in some transgenic pig milk was different, depending on the milking date. (S1 Fig).

**Fig 2:**
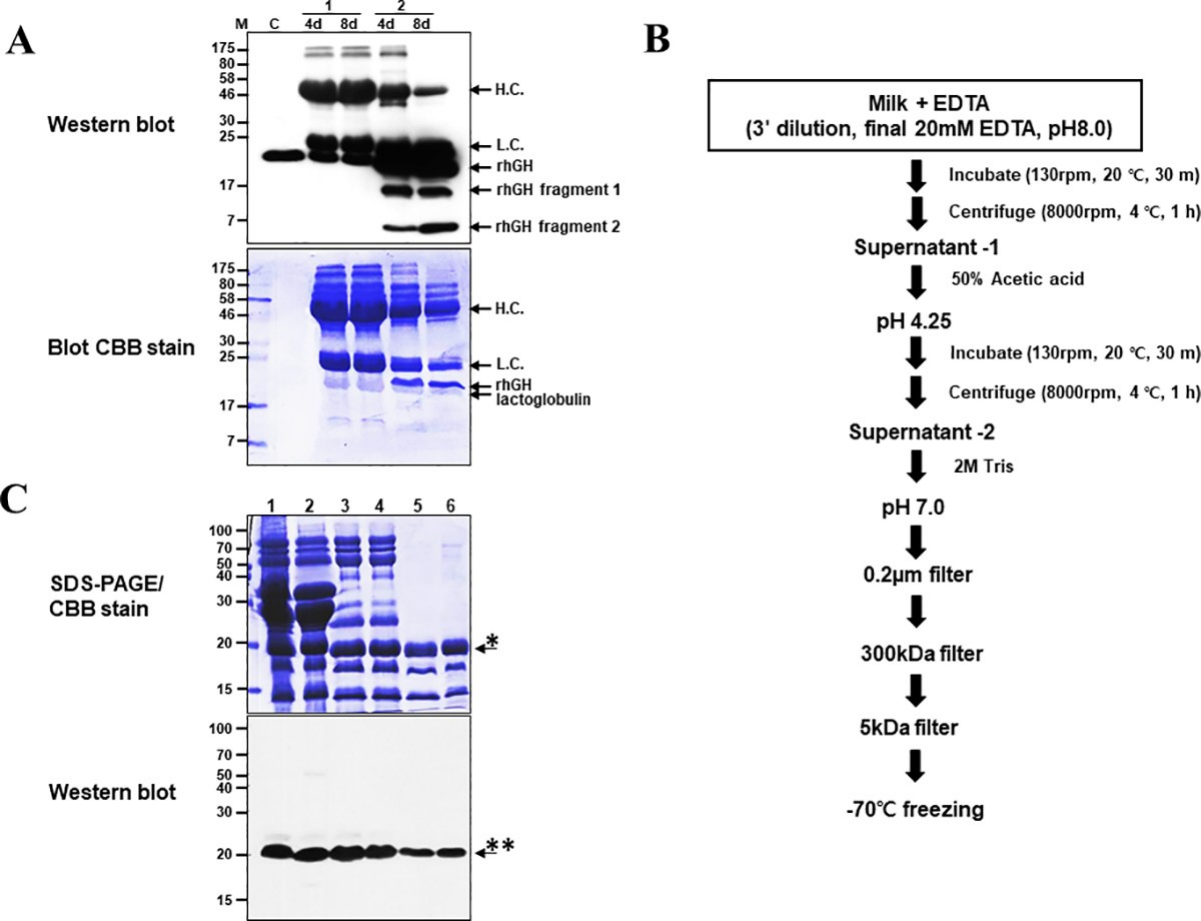
Confirmation of rhGH protein expression in the pig milk and schematics of pretreatment procedures. (A) Confirmation of rhGH protein expression in the pig milk by Western blot assay. Protein samples were loaded onto 13.5% SDS-PAGE gels and analyzed by Western blotting. Then, the blots were stained with Coomassie brilliant blue (CBB). M: marker, C: hGH standard (Ministry of Food and Drug Safety, Korea) 100 μg, 1: rhGH transgenic pig; identification number 68–1 (hGH-LYY 17, F0 generation), 2: rhGH transgenic pig; identification number 94–2 (hGH-LYY 27, F0 generation), d: lactating day, sample loading: crude milk 1 μL, H.C.: Heavy chain, L.C.: Light chain. (B) Schematic of milk pretreatment procedures. (C) CBB staining and Western blotting in different steps of rhGH transgenic porcine milk pretreatment. Protein samples were separated using a 12% SDS-PAGE gel. 1; crude milk, 2; 20 mM EDTA treatment supernatant, 3; 2 M Tris pH 7.4 treatment; 0.2-μm filter permeate, 5; 300-kDa filter permeate, 6; 5-kDa filter retentate. *: impurity (porcine beta- lactoglobulin), **: rhGH.

Since various lipids and proteins were present in the pig milk samples, Coomassie brilliant blue (CBB) staining was insufficient to detect rhGH expression. Thus, Western blotting was performed. Owing to the effect of the secondary antibody, both immunoglobulin heavy and light chains were detected along with rhGH. In the cell lines with the higher expression, rhGH fragments were also detected (Fig 2A). Transgenic pig milk samples showing 0 μg/mL–50 μg/mL rhGH expression were excluded, and the remaining milk samples were collected for rhGH purification. Before purification by chromatography, we removed milk fat and substantial amounts of unwanted proteins. Although we used various milk pretreatment methods described in previous studies, these methods were not able to eliminate casein and larger-sized proteins.

The collected milk was subjected to pretreatment, as shown in Fig 2B, and the protein pattern at each stage of the pretreatment has been outlined in Fig 2C. Although pretreatment allowed a remarkable removal of immunoglobulin and casein, there was substantial remaining lactoglobulin (Fig 2C*), which has a similar protein size as hGH, making it difficult to differentiate the two proteins by CBB staining. Therefore, we planned to remove these proteins along with other impurities during the chromatography purification stage. The amount of milk used in pretreatment was 15.53 L, containing 8.17 g of rhGH, as per sandwich ELISA quantification—the mean rhGH concentration was 526 mg/L. The final collected sample volume was 29.8 L, and the rhGH concentration was 89.74 mg/L, giving an amount of rhGH after pretreatment of 2.68 g, and a final yield of 32.8% (Table 5).

**Table 5:** Yield of transgenic sow milk after pretreatment.

Milk input volume (mL)	15,530
rhGH input (mg)	8177.521
Average rhGH input concentration (μg/mL)	526.56
Milk output volume (mL)	29,890
Average rhGH output concentration (μg/mL)	89.744
rhGH output (mg)^a^	2682.452
Yield (%)^b^	32.8

^a^rhGH output (milligrams) = milk output (milliliter) × average rhGH concentration (micrograms per milliliter).

^b^ Yield (%) = rhGH output (milligrams)/rhGH input (milligrams) × 100.

### Purification of rhGH by column chromatography

**Fig 3:**
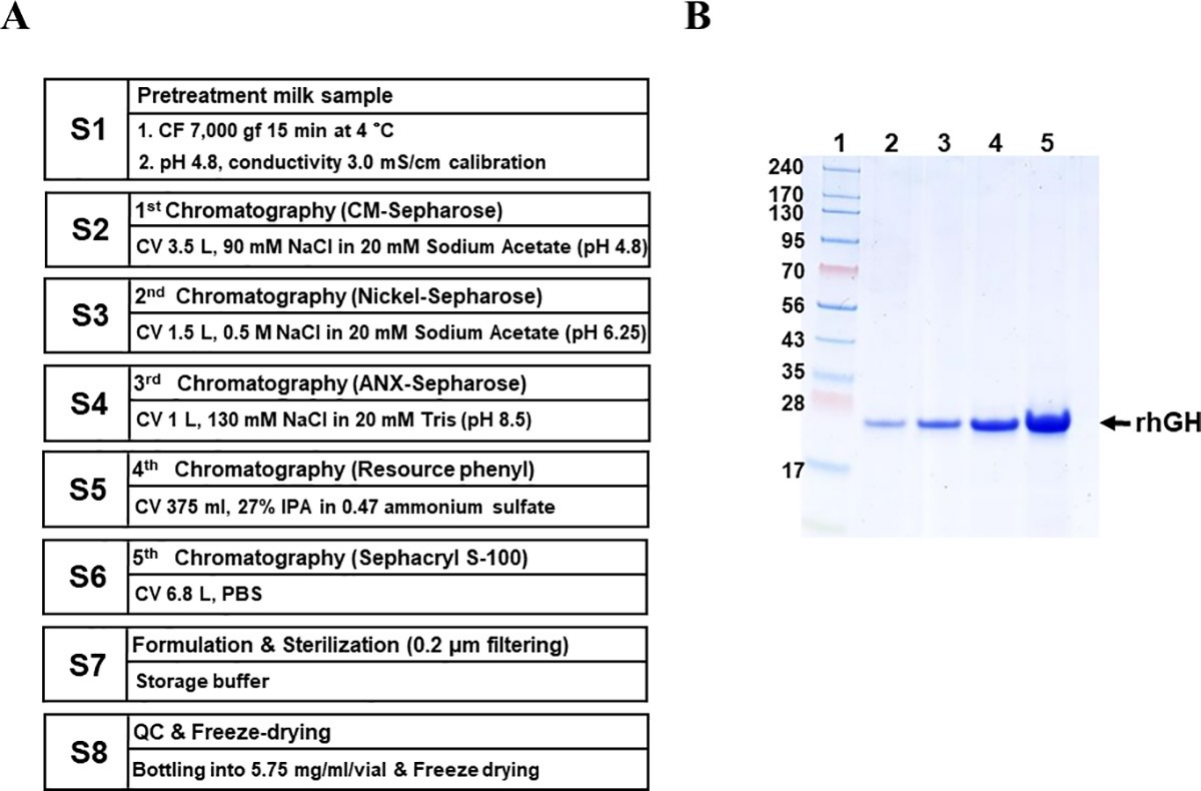
Preclinical sample preparation by column purification. (A) 5-step chromatography and freeze-drying procedure. S1–S8: Step 1 to Step 8 (B) Final purified rhGH was separated using a 5–12% EzWay gel and visualized by CBB staining. 1- Size marker, 2–1 μg, 3–2 μg, 4–5 μg, 5–10 μg.

After pretreatment, several remaining milk proteins had a similar size and pI as those of hGH. To remove the milk proteins that could not be eliminated by the pretreatment process, we followed a 5-step column purification procedure (Fig 3A). rhGH that was cleaved by protease but maintained a similar shape and size as the original protein due to an inner disulfide bond, was also removed by the column purification process. In the input sample used for chromatography purification, the amount of rhGH was ~2.58 g. The amount of final collected protein (rhGH) was ~753 mg. Therefore, the column purification yield was ~29%, and the total yield was ~9.5%. Increasing concentrations of purified rhGH (1, 2, 5, and 10 μg) were loaded onto a 5–12% EzWay (KOMA Biotech, Korea) gel, resolved, and visualized by CBB staining (Fig 3B). The final purified sample was dissolved in the same vehicle used for the commercial drug Genotropin (Pfizer, New York, NY, USA), filtered and lyophilized. Each vial contained 5.75 mg rhGH protein. This product was named as CGH942.

### Characterization of purified rhGH (CGH942)

**Fig 4:**
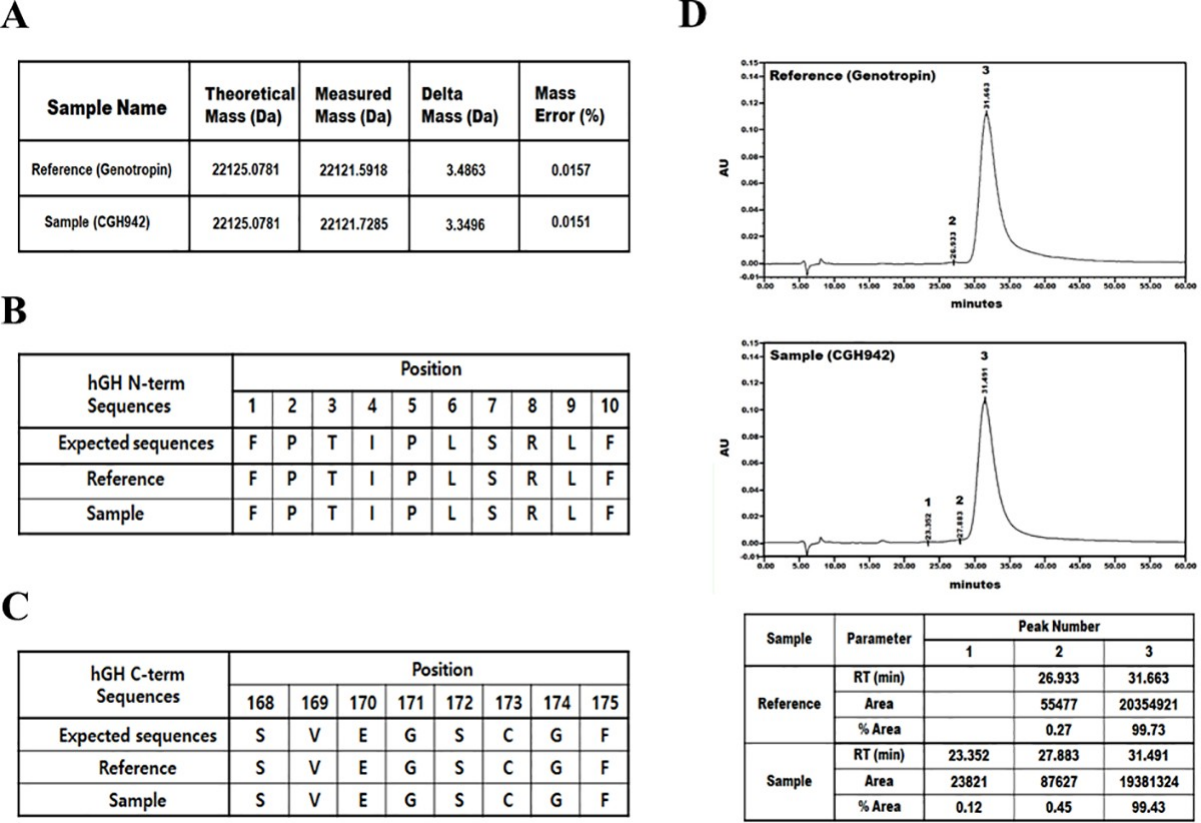
Structural characterization of CGH942. (A) Total mass, (B) N-terminal sequence, (C) C-terminal sequence, and (D) Reverse-phase-UPLC results.

To identify the purified CGH942 protein structure, we performed multiple structural analyses using Genotropin as the control group. Analysis of the total mass by quantitative time-of-flight mass spectrometry indicated that both samples had molecular weights of 22,121.5918–22,121.7285 Da, similar to the molecular weight of hGH. We also calculated a 0.0151%– 0.0157% difference from the expected molecular weight (Fig 4A). N-terminal sequencing was performed using phenylisothiocyanate, which derivatizes N-terminal amino acids. We then analyzed the reaction time of phenylthiohydantoin-amino acid by liquid chromatography to identify the amino acid sequence. A maximum of 10 cycles was analyzed to obtain 10 N-terminal amino acid sequences. The final sequence obtained by N-terminal sequencing was identical to the expected N-terminal amino acid sequence of hGH protein, i.e. ‘NH3- FPTIPLSRLF’ (Fig 4B). C-terminal analysis was performed after cleaving the samples with trypsin and analyzed via reversed-phase ultra-performance liquid chromatography. The extracted ion chromatogram for the expected m/z value of C-terminal peptides was obtained. The C-terminal peptides were detected at ~21 min, and both samples exhibited a mass peak indicating C-terminal peptides with an m/z value of 785.2. Confirmation of the amino acid sequence by MS1/MSE/MS-MS analysis indicated that both samples contained the “SVEGSCGF-COOH” sequence, which is identical to the expected sequence (Fig 4C). Next, we performed reverse-phase high-performance liquid chromatography analysis to assess the relative variant content based on hydrophobicity. Comparative analysis of the area and intensity values for the major and minor peaks in the chromatograms showed that for CGH942, the retention time for peak 3, which was the principal peak, was 31.491 min with a 99.43% area. These values were similar to the reference one (31.663 min retention time with 99.73% area), suggesting that the impurity levels were extremely low (Fig 4D). Similarly, peptide mapping analysis using reverse-phase ultra-performance liquid chromatography (C18) of peptides synthesized after cleavage with trypsin confirmed that the CGH942 and control profiles were identical (S1 Table). Thus, the purified samples contained minimal amounts of impurities and the primary structure of CGH942 was very similar to that of Genotropin.

### Structural characterization of CGH942

**Fig 5:**
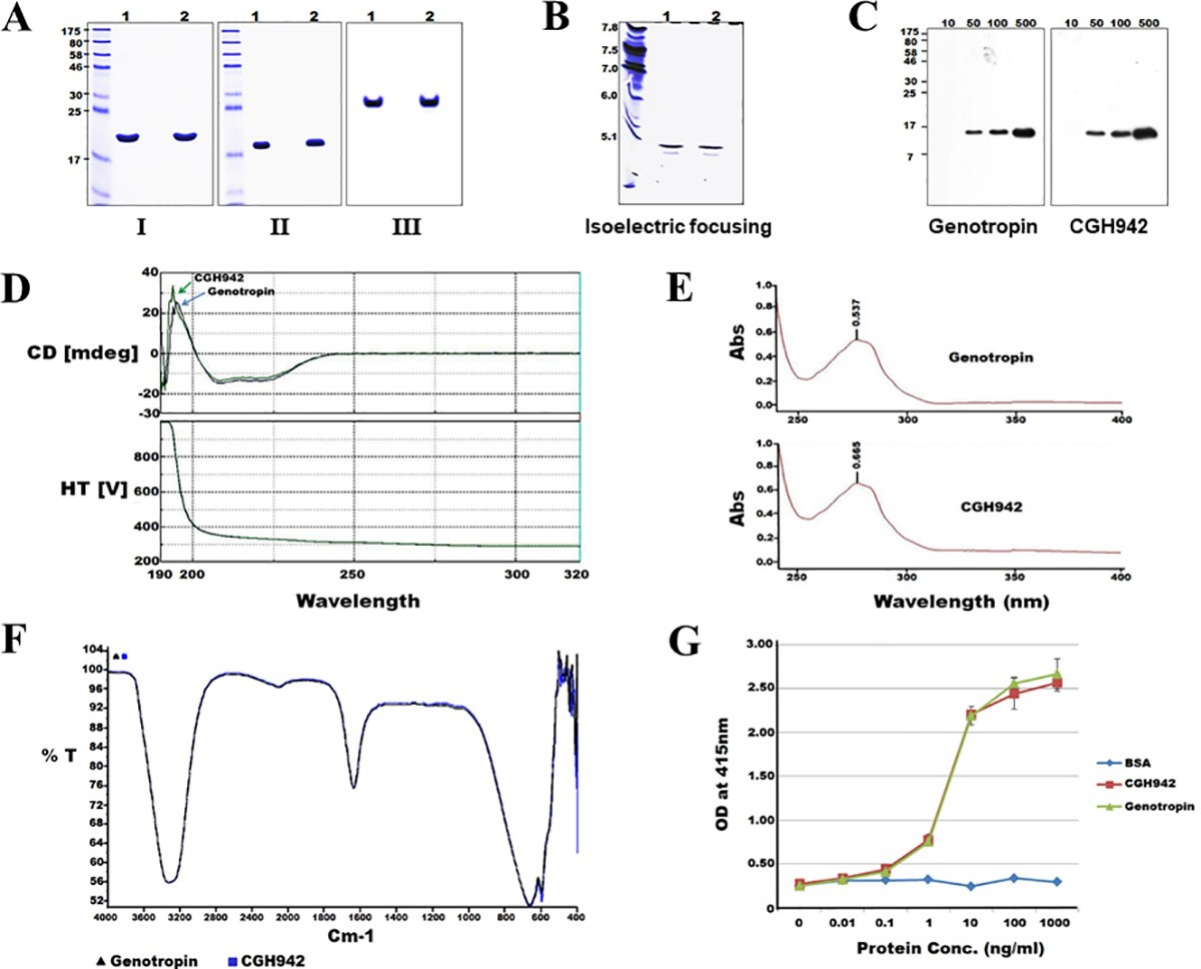
Characterization of purified protein from porcine milk. (A) Electrophoresis I: SDS-PAGE gel (+DTT, reducing), II: SDS-PAGE (-DTT, non-reducing), III: Native gel, 1: Genotropin, 2: CGH942. (B) Isoelectric focusing gel, 1: Genotropin, 2: CGH942. (C) Western blotting. The gel was loaded with 10 ng, 50 ng, 100 ng, and 500 ng rhGH per lane. (D) Circular dichroism spectropolarimeter, HT[V]: High tension voltage. (E) Ultra violet spectrum, Abs: absorbance. (F) Fourier Transform-Infrared (FT-IR) spectrum. (G) Cell proliferation of Nb2-11 cells after rhGH treatments.

Next, we investigated the tertiary structure, efficacy, and presence of modifications via spectroscopic and biological characteristics analyses. First, gel electrophoresis was performed to assess the size and dimerization status. Approximately 5 μg rhGH in the reducing and non-reducing state were loaded onto a 13.5% SDS-PAGE gel. As shown in Fig 5A, rhGH in the reducing state (Fig 5A I) was larger than rhGH in the non-reducing state (Fig 5A II), with the bands located in the same position in native gel electrophoresis (Fig 5A III). These results indicate no difference in the unique characteristics of CGH942 and Genotropin. Moreover, isoelectric focusing showed similarly positioned bands, suggesting that the pI value remained identical after purification (Fig 5B). The antibody affinities of CGH942 and Genotropin were confirmed by Western blotting. 10, 50, 100, and 500 ng/well samples were loaded onto the gel, and the responses for each volume were assessed. As shown in Fig 5C, an identical pattern was observed for both CGH942 and Genotropin. CD spectropolarimetry analysis was performed over wavelengths of 190 nm–320 nm. For both alpha helices and beta turns, neither sample showed a value (0%). The beta sheet ratios were 59.5% and 59.7% and the random coil ratios were 40.5% and 40.3% for the two-dimensional structure of Genotropin and CGH942, respectively, suggesting that there were no structural differences between the two (Fig 5D). Using UV spectrometry, we scanned the prepared samples using UV wavelengths from 220 nm to 400 nm. Both samples exhibited maximum absorbance at 280 nm with identical patterns, suggesting that the two samples were equivalent (Fig 5E). Using Fourier transform-infrared (FT-IR) spectroscopy, we scanned the samples over a range of 4000–400 (cm^-1^) and confirmed the same pattern between Genotropin and CGH942. (Fig 5F).

Further, an i*n vitro* analysis was performed in using Nb2-11 cells, which are lymphoma cells expressing hGH receptors. Biological activity can be measured as proliferation induced by hGH binding to extracellular prolactin receptors. There were no significant differences between CGH942 and Genotropin activities, based on sample concentration (Fig 5G). Therefore, commercially available somatropin (Genotropin) and CGH942 exhibited similar physical and spectroscopic characteristics and biological activities.

### Growth-promoting effects of CGH942 on hypophysectomized SD rats: preclinical tests

**Fig 6:**
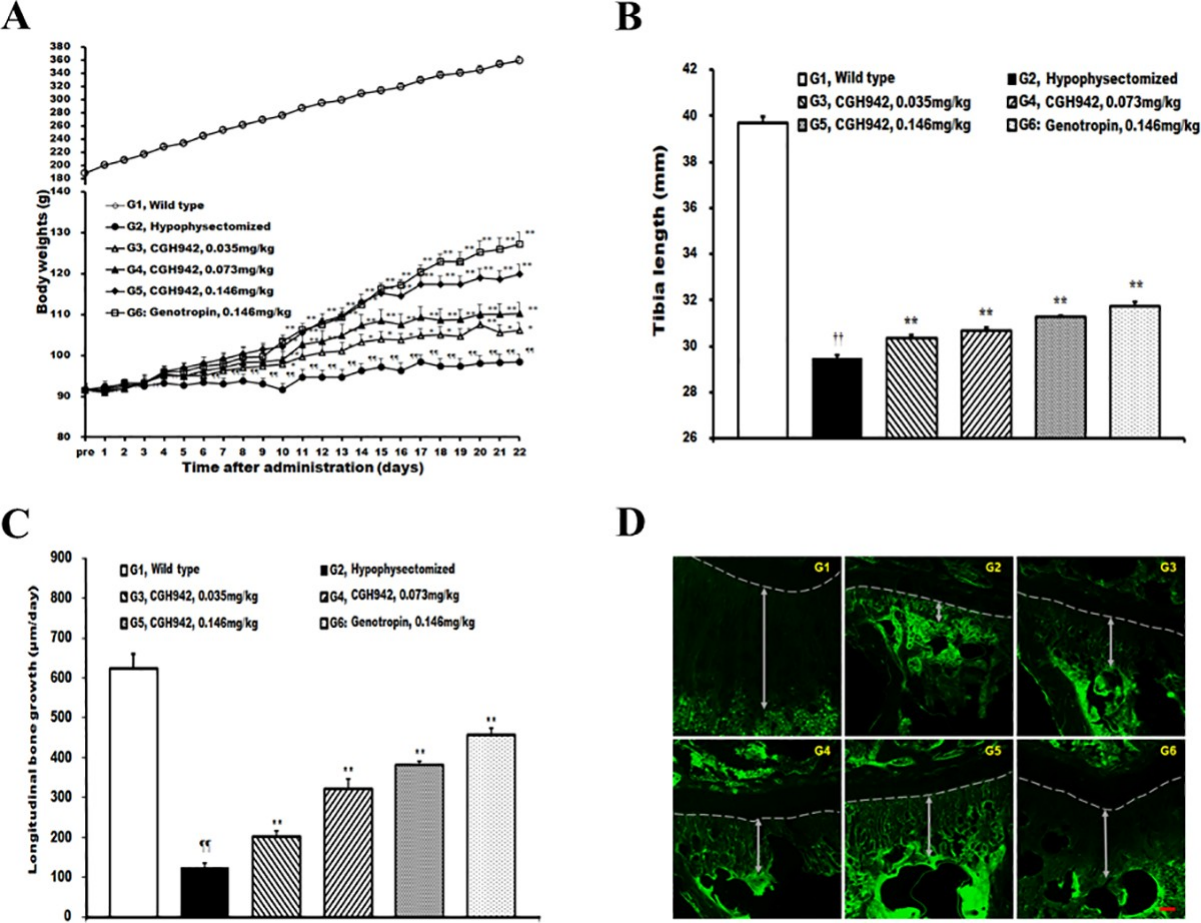
Hypophysectomized rat growth enhancement after subcutaneous CGH942 administration. (A) Bodyweight. Each time point represents the mean ± S.E. (B) Tibia length, (C) Longitudinal bone growth. ^††^*p* < 0.01 compared to the wild type control group (G1) using Student’s *t*-test. ^¶¶^*p* < 0.01 compared to the wild type control group (G1) by the Aspin-Welch *t*-test. *p < 0.05 compared to the hypophysectomized control group (G2) by the Dunnett's *t*-test. **p < 0.01, compared to the hypophysectomized control group (G2) by the Dunnett's *t*-test. (D) Fluorescent photomicrographs of the proximal tibia growth plate longitudinal sections, G1: Wild type control; G2: Hypophysectomized control; G3: CGH942 0.035 mg/kg; G4: CGH942 0.076 mg/kg; G5: CGH942 0.146 mg/kg; G6: Genotropin 0.146 mg/kg. Double head arrows indicate the distance between the growth plate and the band formed by tetracycline. ×50, Scale bar = 100 μm.

Although the molecular and *in vitro* biological characteristics of CGH942 protein were similar to those of Genotropin, we performed additional non-clinical tests to validate the CGH942 effectiveness *in vivo* and assess its potential toxicity for human use. First, we performed repeated subcutaneous injections to hypophysectomized male rats to assess the effectiveness of CGH942. We hypothesized that administration of CGH942 would rescue the dwarfism caused by pituitary gland removal. Thus, we measured the rats’ weight, tibia length, and bone growth rate. The weight of rats in the hypophysectomized control group was significantly reduced on all assessment dates compared to the wild type control group. Throughout the experimental period, the mean weight gain was 6.0 g, indicating that removal of the pituitary gland caused growth retardation. The group injected with 0.035 mg/kg CGH942 (G3) exhibited a significant weight increase 13 days after injection compared to the hypophysectomized control group. The groups injected with 0.073 mg/kg CGH942 (G4), 0.146 mg/kg CGH942 (G5) and with 0.146 mg/kg Genotropin (G6) all exhibited significant increases 10 days after injection. All groups exhibited dose-dependent weight gain (Fig 6A). The hypophysectomized control group exhibited a significantly reduced tibia length compared to that in the wild type control group. Importantly, all animals that received CGH942 exhibited significant dose-dependent increased tibia length compared to that of the hypophysectomized control group. Similarly, the group injected with Genotropin showed a significantly increased tibia length compared to the hypophysectomized control group (Fig 6B and 6D). Assessment of the bone growth rate showed similar findings, also in a dose-dependent fashion (Fig 6C). In conclusion, administration of CGH942 to rats with growth retardation (induced by removal of the pituitary gland) promoted bone growth and weight gain, suggesting that CGH942 promoted an overall growth.

### Toxicity and toxicokinetics of CGH942

Safety assessment was performed by repeated subcutaneous injection of CGH942 into male and female rats for 4 weeks. The reversibility of any effects was assessed after a 2-week recovery period. Furthermore, toxicokinetic tests were performed to evaluate systemic exposure to CGH942 or Genotropin. In the entire toxicity study, there were no deaths or abnormal symptoms induced by CGH942. Further, toxicity was not observed in most examinations or tests. Histopathological examination indicated the presence of mixed inflammatory cell infiltration at the injection site in all rhGH-injected groups, but all rats recovered after 2 weeks. Considering that the ingredients in CGH942 and Genotropin are human-derived proteins, this change may reflect the normal response mounted to foreign substances and was not toxicologically significant. On injection day 1, systemic somatropin levels (in the bloodstream; AUCl_ast_ and C_max_) tended to increase in proportion to the injection amount (1:2:4) in both male and female groups, with no significant difference comparing CGH942 and Genotropin. The AUC_last_ and C_max_ values for both CGH942 and Genotropin were 1.6–3.8-fold higher in the males than in females, revealing a sex-based difference. On day 28 after injection, systemic somatropin in the bloodstream could not be accurately assessed because of the large variances among individual animals. The time to reach the peak plasma concentration (T_max_) was 0.3–0.5 h on Day 1 and 0.5–3.0 h on Day 28 (Table 6). The findings from multiple assessments of CGH942 toxicity suggested that the no-observed-adverse-effect-level of CGH942 was 0.6 mg/kg/day for both males and females. Importantly, there was no significant toxicological difference between CGH942 and Genotropin.

**Table 6:** Toxicokinetic parameters of CGH942 and Genotropin in rats.

Sex	Group/Dose (mg/kg/day)	Phase	Toxicokinetic parameters		
			AUC_last_ (ng·h/mL)	C_max_ (ng·h/mL)	T_max_ (h)
Male	G1 / 0.15	Day 1	45.5	40.2	0.5
		Day 28	60.7	22.1	0.5
	G2 / 0.3	Day 1	99.3	54.6	0.5
		Day 28	0.2	0.9	1.0
	G3 / 0.6	Day 1	226.4	121.9	0.3
		Day 28	123.8	23.3	1.0
	G4 / 0.6	Day 1	272.8	230.3	0.5
		Day 28	79.5	11.9	1.0
Female	G1 / 0.15	Day 1	11.9	15.6	0.3
		Day 28	33.2	7.0	3.0
	G2 / 0.3	Day 1	55.7	33.4	0.5
		Day 28	6.8	4.4	1.0
	G3 / 0.6	Day 1	121.3	64.6	0.5
		Day 28	194.4	49.3	1.0
	G4 / 0.6	Day 1	137.5	109.7	0.3
		Day 28	178.3	27.5	3.0

## Discussion

The present study aimed to assess the potential of transgenic pigs as bioreactors for the production of therapeutic proteins. Various attempts have been made to produce biologically active recombinant proteins in transgenic pig milk [31–33]. However, no follow-up studies toward mass production have been published. Here, we used transgenic pigs to mass-produce a biologically active heterologous protein and confirmed its efficacy and safety. Our study showed lower cleavage and blastocyst production rates after SCNT compared with a previous report on the production of transgenic pigs [34]. This suggests our embryo development rates can be improved, which we plan to pursue in a follow-up study. Although our transgenic offspring rate was lower than that published in a previous report [33], we remarkably still produced 69 transgenic pigs and confirmed germline transmission. In order to avoid inbreeding (thinking on a long-term perspective), we established different transfected cell lines by securing the fetal tissues and used them to produce different progenies. Among the produced transgenic pigs, those with exceptionally high rhGH expression were selected for AI to obtain an adequate number of transgenic pigs. This suggests the possibility of potential scale-up of rhGH production from transgenic pig milk. A previous report suggested that AI results in 7.3 piglets produced in the first parturition, with the number increasing in the second and third parturitions [35]. Although our study exhibited good outcomes, the number of piglets showed a tendency to decrease in subsequent pregnancies. In addition, although the rate of germline transmission increased in subsequent pregnancies, further studies are needed to evaluate this outcome.

Recombinant hGH was expressed in different levels, ranging from 7.8 μg/mL to 1.7 mg/mL in the collected transgenic pigs’ milk. Furthermore, the rhGH concentration in milk collected daily showed a variation depending on the lactating days. In general, colostrum contains less lactose and more lipids, proteins, carbohydrates, vitamins, growth factors, enzymes, enzyme inhibitors, cytokines and minerals than mature milk [36–38]. In contrast, the level of rhGH in early post-partum milk samples increased in milk after colostrum. Therefore, we hypothesize that the milk composition may affect rhGH production over the lactation period. However, rhGH concentration did not steadily increase or decrease; various expression tendencies were shown, as can be seen in S1 Fig. We inferred that these differences could be attributed to the nutrition and biorhythm of each pig during the lactation period. Some previous studies [33, 39] have reported that recombinant proteins expressed in transgenic pig milk vary depending on the date of lactation, but the mechanism behind was not explained. Therefore, more research is needed to understand how lactation impacts recombinant protein expression. Furthermore, depending on the transgenic PFF cell line used as donor cells, the rhGH expression levels in transgenic pig milk samples were different. The difference in expression based on individual cell lines may be related to the gene transfection method used in our study. We performed gene transfection into somatic cells by random insertion but did not confirm the integration site or copy numbers. Transgene integration was confirmed using PCR at the genomic level after the selection of single cell colonies. Thus, by crossbreeding transgenic pigs with high rhGH yield and pigs with exceptionally high milk production, the transgenic pig quality would likely be improved. Future studies will confirm the correlation between transgene copy number and integration site and recombinant protein secretion.

The liquid chromatography results indicated that CGH942 with impurities < 1% was obtained from the purification process, suggesting that the purified protein is suitable for clinical usage. In general, milk is often pre-treated with zinc chloride to remove as much casein and whey protein as possible [40]. However, because zinc ion induces hGH dimerization [41], this process could not be utilized in our study. Moreover, experiments using calcium phosphate particles (CAP) [42], citric acid [43], and sodium phosphate [44] were explored for casein removal from the milk of transgenic animals; however, casein was not completely removed. After several trials and various experiments, we established the pretreatment process outlined in Fig 2B. Nevertheless, there were substantial impurities remaining after the process, including rhGH fragments digested by tryptic peptidases [45–47]. Therefore, it was challenging to produce high-purity rhGH by traditional one or two-stage column chromatography purification methods used e.g. for *E*. *coli* [48–50]. We established a 5-step column purification process to obtain high-purity rhGH but the purification yield of this process was not satisfactory. There have been several reports on the purification of rhGH from the milk of transgenic animals [21–22]. However, the methods reported in these studies, depending mostly on affinity columns (his-tag or immune-based), could be not applied in this study. Therefore, additional studies should focus on simplifying the pretreatment process and improving the final yield of rhGH purified from pigs’ milk.

Although we were able to successfully obtain biologically active rhGH from transgenic pigs’ milk, the selection of suitable livestock species to produce target proteins should be carefully (and rationally) performed. As a bioreactor (in this context), animal should be able to produce large amounts of milk during the entire lactation periods; furthermore, the feasibility and the breeding/housing costs should also be considered [51]. Many proteins require post-translational modifications such as signal peptide removal, disulfide bonds formation, amino acid modifications, and proteolytic processing. Glycosylation and carboxylation are essential for the biological activity and/or stability of many proteins and are key factors for the production of biologically active pharmaceuticals by recombinant organisms [51]. In fact, some recombinant glycoproteins produced using transgenic pigs were reported to have different sugar moieties, compared with the human-derived ones [31, 52, 53]. Overall, for the production of therapeutic recombinant proteins, we need to ensure either the glycosylation patterns are similar to the original ones, or that potential changes do not affect protein activity.

Although previous studies have demonstrated the feasibility of using transgenic animals traditionally associated with high milk yields (i.e., cows or goats) for bio-drug production, no studies have confirmed these results in non-dairy animals such as pigs. In addition to the 15.5 L of pig milk produced for non-clinical sample production, we collected ~140 L of additional pig milk, indicating that pigs can function as bioreactors with a sufficient milk supply for mass production of protein-based drugs.

## Conclusion

This study confirmed the efficacy and safety profiles of rhGH produced in transgenic pigs are equivalent to those of commercial somatropin. Our outcomes have suggested that transgenic pigs are suitable for large-scale protein-based drug production.

## Supporting information

S1 Table Peptide map analyses of rhGH protein samples. (DOCX)

S1 Fig Verification of rhGH protein expression in milk collected across the lactation period. A) Total milk protein separation was assessed by Coomassie brilliant blue (CBB) staining. rhGH protein expression was confirmed by Western blot assay. rhGH TG1: rhGH transgenic pig identification number 39–3 (rhGH-LYY 17, F0 generation). The lactation period was 40 days. Protein samples were loaded onto 12% SDS-PAGE gels after 10X dilution in distilled water. After Western blot assays, the same gel was stained with CBB. Crude milk loading volume: 0.5 μL. Primary antibody treatment 1:3000, secondary antibody treatment 1:5000. rhGH TG2: rhGH transgenic pig identification number 94–2 (hGH-LYY 27, F0 generation). The lactation period was 35 days. Protein samples were loaded onto 13.5% SDS-PAGE gels after 10–20X dilution in distilled water. Crude milk loading volume: SDS-PAGE: 0.5 μL, Western blot: 0.1 μL. Primary antibody treatment 1:5000, secondary antibody treatment 1:10000. (B) Quantification of rhGH protein expression by ELISA assay during lactation period.
(TIF)

## References

[1] Lewis U, Bonewald L, Lewis LJ. The 20,000-dalton variant of human growth hormone: location of the amino acid deletions. Biochemical and Biophysical Research Communications. 1980;92(2):511–6. 10.1016/0006-291x(80)90363-0 7356479

[2] Lewis UJ, Peterson S, Bonewald L, Seavey B, VanderLaan WP. An interchain disulfide dimer of human growth hormone. The Journal of Biological Chemistry. 1977;252(11):3697–702. 863899

[3] Dienys G, Sereikaitė J, Lukša V, Jarutienė O, Mištinienė E, Bumelis V-A. Dimerization of human growth hormone in the presence of metal ions. Bioconjugate Chemistry. 2000;11(5):646–51. 10.1021/bc990112j 10995207

[4] Goeddel DV, Heyneker HL, Hozumi T, Arentzen R, Itakura K, Yansura DG, et al Direct expression in Escherichia coli of a DNA sequence coding for human growth hormone. Nature. 1979;281(5732):544 10.1038/281544a0 386136

[5] Chang CN, Key M, Bochner B, Heyneker H, Gray G. High-level secretion of human growth hormone by Escherichia coli. Gene. 1987;55(2):189–96. 10.1016/0378-1119(87)90279-4 3311882

[6] Fahnert B, Lilie H, Neubauer P. Inclusion bodies: formation and utilisation. Physiological Stress Responses in Bioprocesses: Springer; 2004 p. 93–142. 10.1007/b93995 15217157

[7] Ikehara M, Ohtsuka E, Tokunaga T, Taniyama Y, Iwai S, Kitano K, et al Synthesis of a gene for human growth hormone and its expression in Escherichia coli. Proceedings of the National Academy of Sciences. 1984;81(19):5956–60. 10.1073/pnas.81.19.5956 6091124 PMC391837

[8] Franchi E, Maisano F, Testori SA, Galli G, Toma S, Parente L, et al A new human growth hormone production process using a recombinant Bacillus subtilis strain. Journal of biotechnology. 1991;18(1–2):41–54. 10.1016/0168-1656(91)90234-m 1367506

[9] Catzel D, Lalevski H, Marquis CP, Gray PP, Van Dyk D, Mahler SM, et al Purification of recombinant human growth hormone from CHO cell culture supernatant by Gradiflow preparative electrophoresis technology. Protein Expression and Purification. 2003;32(1):126–34. 10.1016/j.pep.2003.07.002 14680949

[10] Geng Z-H, Liu Y, Gao P, Zhao D, Li S, Yu X, et al Enhancing hGH expression level in insect cells by shortening the 5'-UTR of hGH cDNA. Sheng Wu Gong Cheng Xue Bao. 2002;18(4):505–8. 12385253

[11] Ascacio-Martínez JA, Barrera-Saldaña HA. Production and secretion of biologically active recombinant canine growth hormone by Pichia pastoris. Gene. 2004;340(2):261–6. 10.1016/j.gene.2004.06.058 15475167

[12] Maksimenko O, Deykin A, Georgiev PG. Use of transgenic animals in biotechnology: prospects and problems. Acta Naturae 2013;5(1 (16)). 10.32607/20758251-2013-5-1-33-46 PMC3612824 23556129

[13] Hammer RE, Brinster RL, Rosenfeld MG, Evans RM, Mayo KE. Expression of human growth hormone-releasing factor in transgenic mice results in increased somatic growth. Nature. 1985;315(6018):413 10.1038/315413a0 3923368

[14] Wilmut I, Schnieke AE, McWhir J, Kind AJ, Campbell KH. Viable offspring derived from fetal and adult mammalian cells. Nature. 1997;385(6619):810 10.1038/385810a0 9039911

[15] Echelard Y, Williams JL, Destrempes MM, Koster JA, Overton SA, Pollock DP, et al Production of recombinant albumin by a herd of cloned transgenic cattle. Transgenic Research. 2009;18(3):361–76. 10.1007/s11248-008-9229-9 19031005

[16] Whyte JJ, Prather RS. Genetic modifications of pigs for medicine and agriculture. Molecular Reproduction & Development. 2011;78(10‐11):879–91. 10.1002/mrd.21333 21671302 PMC3522184

[17] Van Cott KE, Butler SP, Russell CG, Subramanian A, Lubon H, Gwazdauskas F, et al Transgenic pigs as bioreactors: a comparison of gamma-carboxylation of glutamic acid in recombinant human protein C and factor IX by the mammary gland. Genetic Analysis: Biomolecular Engineering. 1999;15(3–5):155–60. 10.1016/s1050-3862(99)00020-0 10596756

[18] Korhonen VP, Tolvanen M, Hyttinen JM, Uusi-Oukari M, Sinervirta R, Alhonen L, et al Expression of bovine beta-lactoglobulin/human erythropoietin fusion protein in the milk of transgenic mice and rabbits. European journal of biochemistry. 1997;245(2):482–9 10.1111/j.1432-1033.1997.00482.x 9151983

[19] Kwon D-N, Song H, Park J-Y, Lee S-Y, Cho S-K, Kang S-J, et al Dynamic control of oligosaccharide modification in the mammary gland: linking recombinant human erythropoietin. Transgenic Research. 2006;15(1):37–55. 10.1007/s11248-005-3519-2 16475009

[20] Lee C-S, Lee DS, Fang N-Z, Oh KB, Shin S-T, Lee K-K, et al Integration and Expression of Goat B-CaseinlhGH Hybrid Gene in a Transgenic Goat. Reproductive & developmental biology. 2006;30(4):293–9. https://www.earticle.net/Article/A32264

[21] Salamone D, Barañao L, Santos C, Bussmann L, Artuso J, Werning C, et al High level expression of bioactive recombinant human growth hormone in the milk of a cloned transgenic cow. Journal of biotechnology. 2006;124(2):469–72. 10.1016/j.jbiotec.2006.01.005 16716426

[22] Lipinski D, Zeyland J, Szalata M, Plawski A, Jarmuz M, Jura J, et al Expression of human growth hormone in the milk of transgenic rabbits with transgene mapped to the telomere region of chromosome 7q. Journal of Applied Genetics. 2012;53(4):435–42. 10.1007/s13353-012-0110-4 22898896 PMC3477484

[23] Kim JH, Yeo MG, Kang S-J, Ahn JD. Gene of porcine beta casein, a promoter of the same and the use thereof. Google Patents; 2013, US8420388B2.

[24] Potter H, Weir L, Leder P. Enhancer-dependent expression of human kappa immunoglobulin genes introduced into mouse pre-B lymphocytes by electroporation. Proceedings of the National Academy of Sciences. 1984;81(22):7161–5. 10.1073/pnas.81.22.7161 6438633 PMC392097

[25] Park K-W, Lai L, Cheong H-T, Im G-S, Sun Q-Y, Wu G, et al Developmental potential of porcine nuclear transfer embryos derived from transgenic fetal fibroblasts infected with the gene for the green fluorescent protein: comparison of different fusion/activation conditions. Biology of reproduction. 2001;65(6):1681–5. 10.1095/biolreprod65.6.1681 11717128

[26] Wang W, Abeydeera L, Cantley T, Day BN. Effects of oocyte maturation media on development of pig embryos produced by in vitro fertilization. Journal of reproduction fertility. 1997;111(1):101–8. 10.1530/jrf.0.1110101 9370973

[27] Wang WH, Abeydeera LR, Prather RS, Day BN. Functional analysis of activation of porcine oocytes by spermatozoa, calcium ionophore, and electrical pulse. Molecular Reproduction and Development. 1998;51(3):346–53. 10.1002/(SICI)1098-2795(199811)51:3&lt;346::AID-MRD15&gt;3.0.CO;2-0 9771656

[28] Yin XJ, Cho SK, Park MR, Im YJ, Park JJ, Jong Sik B, et al Nuclear remodelling and the developmental potential of nuclear transferred porcine oocytes under delayed-activated conditions. Zygote (Cambridge, England). 2003;11(2):167–74. 10.1017/s0967199403002 20x. 12828416

[29] Quilter CR, Blott SC, Mileham AJ, Affara NA, Sargent CA, Griffin DK. A mapping and evolutionary study of porcine sex chromosome gene. Mammalian Genome. 2002;13(10):588–94. 10.1007/s00335-002-3026-1 12420137

[30] Lee JM, Lee MA, Do HN, Bae RJN, Lee MJ, Kim MJ, et al Historical Control Data from 4-week Repeated Toxicity Studies in Crj:CD (SD) Rats. Lab Anim Res. 2012;28(2):115–21. 10.5625/lar.2012.28.2.115 22787485 PMC3389835

[31] Park JK, Lee YK, Lee P, Chung HJ, Kim S, Lee HG, et al Recombinant human erythropoietin produced in milk of transgenic pigs. Journal of biotechnology. 2006;122(3):362–71. 10.1016/j.jbiotec.2005.11.021 16460825

[32] Lee HG, Lee HC, Kim SW, Lee P, Chung HJ, Lee YK, et al Production of recombinant human von Willebrand factor in the milk of transgenic pigs. The Journal of reproduction and development. 2009;55(5):484–90. 10.1262/jrd.20212 19521054

[33] Lu D, Liu S, Shang S, Wu F, Wen X, Li Z, et al Production of transgenic-cloned pigs expressing large quantities of recombinant human lysozyme in milk. PLoS ONE. 2015;10(5): e0123551 10.1371/journal.pone.0123551 25955256 PMC4425539

[34] Park KW, Cheong HT, Lai L, et al Production of nuclear transfer-derived swine that express the enhanced green fluorescent protein. Anim Biotechnol. 2001;12(2):173–181. 10.1081/abio-100108344 11808633

[35] Holtz W, Schmidt-Baulain R, Welp C, Wallenhorst Chr.K. Effect of insemination of estrus-induced prepuberal gilts on ensuing reproductive performance and body weight. Animal reproduction science. 1999;57(3–4):177–83. 10.1016/s0378-4320(99)00054-8 10610037

[36] Parrish D, Wise G, Hughes J, Atkeson F.W. Properties of the Colostrum of the Dairy Cow. V. Yield, Specific Gravity and Concentrations of Total Solids and its Various Components of Colostrum and Early Milk. Journal of Dairy Science. 1950;33(6):457–65. 10.3168/jds.S0022-0302(50)91921-7

[37] Elfstrand L, Lindmark-Månsson H, Paulsson M, Nyberg L, Åkesson BJ. Immunoglobulins, growth factors and growth hormone in bovine colostrum and the effects of processing. International Dairy Journal. 2002;12(11):879–87. 10.1016/S0958-6946(02)00089-4

[38] Brian A. M., Patrick F. F., Paul L. H. M., Alan L. K. Composition and properties of bovine colostrum: a review. Dairy Sci. & Technol. 2016; 96:133–158 10.1007/s13594-015-0258-x

[39] Mukherjee A, Garrels W, Talluri TR, Tiedemann D, Bősze Z, Ivics Z, et al Expression of active fluorophore proteins in the milk of transgenic pigs bypassing the secretory pathway. scientific reports. 2016;6(1):1–10. 10.1038/s41598-016-0001-8 27086548 PMC4834472

[40] Mohanty D, Mohapatra S, Misra S, Sahu PJ. Milk derived bioactive peptides and their impact on human health–A review. Saudi journal of biological sciences. 2016;23(5):577–83. 10.1016/j.sjbs.2015.06.005 27579006 PMC4992109

[41] Cunningham BC, Mulkerrin MG, Wells JAJ. Dimerization of human growth hormone by zinc. Science. 1991;253(5019):545–8. 10.1126/science.1907025 1907025

[42] Morçöl T, He Q, Bell SJD. Model process for removal of caseins from milk of transgenic animals. Biotechnology progress. 2001;17(3):577–82. 10.1021/bp010023x 11386883

[43] Pan K, Zhong Q. Improving clarity and stability of skim milk powder dispersions by dissociation of casein micelles at pH 11.0 and acidification with citric acid. Journal of agricultural and food chemistry. 2013;61(38):9260–8. 10.1021/jf402870y 23985027

[44] Yen C-H, Lin Y-S, Tu C-F, Biotechnology. A Novel Method for Separation of Caseins from Milk by Phosphates Precipitation. Preparative Biochemistry and Biotechnology 2015;45(1):18–32. 10.1080/10826068.2013.877030 24372141

[45] Witkowska E, Orłowska A, Sagan B, Smoluch M, Izdebski J. Tryptic hydrolysis of hGH‐RH (1‐29) ‐NH2 analogues containing Lys or Orn in positions 12 and 21. Journal of peptide science. 2001;7(3):166–72. 10.1002/psc.316 11297353

[46] WROBLEWSKI VJ, MASNYK M, BECKER GW. Proteolytic cleavage of human growth hormone (hGH) by rat tissues in vitro: influence on the kinetics of exogenously administered hGH. Endocrinology. 1991;129(1):465–74. 10.1210/endo-129-1-465 2055201

[47] Komatsu N, Saijoh K, Otsuki N, Kishi T, Micheal IP, Obiezu CV, et al Proteolytic processing of human growth hormone by multiple tissue kallikreins and regulation by the serine protease inhibitor Kazal-Type5 (SPINK5) protein. Clinica Chimica Acta. 2007;377(1–2):228–36. 10.1016/j.cca.2006.10.009 17140555

[48] Mukhija R, Rupa P, Pillai D, Garg LC. High-level production and one-step purification of biologically active human growth hormone in Escherichia coli. Gene. 1995;165(2):303–6. 10.1016/0378-1119(95)00525-b 8522194

[49] Patra AK, Mukhopadhyay R, Mukhija R, Krishnan A, Garg L, Panda AK, et al Optimization of inclusion body solubilization and renaturation of recombinant human growth hormone from Escherichia coli. Protein Expression and Purification. 2000;18(2):182–92. 10.1006/prep.1999.1179 10686149

[50] de Oliveira JE, Soares CR, Peroni CN, Gimbo E, Camargo IM, Morganti L, et al High-yield purification of biosynthetic human growth hormone secreted in Escherichia coli periplasmic space. Jounal of Chromatography A. 1999;852(2):441–50. 10.1016/S0021-9673(99)00613-5 10481982

[51] Bösze Z, Baranyi M, Bruce C, Whitelaw A. Producing recombinant human milk proteins in the milk of livestock species. Bioactive Components of Milk. Springer, 2008; p 357–95. 10.1007/978-0-387-74087-4_15 18183938

[52] Lee M-H, Lin Y-S, Tu C-F, Yen C-HJ. Recombinant human factor IX produced from transgenic porcine milk. Biomed Res Int. 2014;2014 10.1155/2014/315375 PMC4052152 24955355

[53] Velander WH, Johnson JL, Page RL, Russell CG, Subramanian A, Wilkins TD, et al High-level expression of a heterologous protein in the milk of transgenic swine using the cDNA encoding human protein C. Proc Natl Acad Sci U S A. 1992;89(24):12003–7. 10.1073/pnas.89.24.12003 1465430 PMC50686

