# Supplementary material for: Structural and functional characterization of recombinant human growth hormone isolated from transgenic pig milk
Source: PLoS One. 2020 Jul 31;15(7):e0236788. doi: 10.1371/journal.pone.0236788 (PMC7394428; doi:10.1371/journal.pone.0236788)
Supplement: S1 Table — (DOCX) [file pone.0236788.s001.docx]

Supporting information

**Structural and functional characterization of recombinant human growth hormone isolated from milk of transgenic pig milk**

*So-Young Lee^1^*^¶^, Joo-Hee Han^1,2¶^, Eun-Kyeong Lee^1^, Young Kyu Kim^1^, Seo-Ah Hwang ^1^, Sung-Hyun Lee^1^, Maria Kim^1^, Gye Yoon Cho ^1^, Jae-Ha Hwang^1^, Su-Jin Kim^1^, Jae-Gyu Yoo^3^, Seong-Keun Cho^2^, Kyung-Ju Lee^1^ and Weon-Ki Cho^1^*

So-Young Lee

E-mail: isso97@choa.co.kr

S1 Table

**S1 Table. Peptide map analysis of rhGH protein samples**

| **Peptide** | **Fragment Number** | **Calculated Peptide  Mass (Da)** | **Reference (Genotropin Z07139)** | | **Sample (CGH942)** | |
| --- | --- | --- | --- | --- | --- | --- |
|  |  |  | **RT (Min)** | **Mass (Da)** | **RT (Min)** | **Mass (Da)** |
| FPTIPLSR | 1:T001 | 929.5334 | 31.6 | 929.4428 | 31.6 | 929.4389 |
| LFDNAMLR | 1:T002 | 978.4957 | 29.5 | 978.4064 | 29.5 | 978.399 |
| AHR | 1:T003 | 382.2077 | 3.8 | 382.1713 | 3.8 | 382.1702 |
| LHQLAFDTYQEFEEAYIPK | 1:T004 | 2341.1267 | 48.7 | 2340.9128 | 48.7 | 2340.9163 |
| LHQLAFDTYQEFEEAYIPKEQK | 1:T004-005 | 2726.3228 | 47 | 2726.092 | 47 | 2726.0774 |
| EQK | 1:T005 | 403.2067 | 2.2 | 403.1684 | 2.2 | 403.1679 |
| YSFLQNPQTSLCFSESIPTPSNR | 1:T006 | 2615.2327 | 47.7 | 2615.0027 | 47.7 | 2614.9993 |
| YSFLQNPQTSLCFSESIPTPSNRE  ETQQK | 1:T006-007 | 3358.5776 | 46.8 | 3358.3015 | 46.8 | 3358.2751 |
| EETQQK | 1:T007 | 761.3555 | 5.8 | 761.2849 | 5.8 | 761.2852 |
| SNLELLR | 1:T008 | 843.4814 | 25.4 | 843.4049 | 25.3 | 843.3976 |
| ISLLLIQSWLEPVQFLR | 1:T009 | 2054.1929 | 62.2 | 2054.0132 | 62.2 | 2054.0095 |
| SVFANSLVYGASDSNVYDLLK | 1:T010 | 2261.1216 | 50.6 | 2260.9207 | 50.6 | 2260.9148 |
| DLEEGIQTLMGR | 1:T011 | 1360.6656 | 46.6 | 1360.5355 | 46.6 | 1360.5306 |
| LEDGSPR | 1:T012 | 772.3715 | 11.1 | 772.2985 | 11.1 | 772.2954 |
| TGQIFK | 1:T013 | 692.3857 | 13.8 | 692.3196 | 13.8 | 692.3169 |
| QTYSK | 1:T014 | 625.3071 | 8 | 625.2466 | 8 | 625.2442 |
| FDTNSHNDDALLK | 1:T015 | 1488.6844 | 21.6 | 1488.545 | 21.6 | 1488.5378 |
| FDTNSHNDDALLKNYGLLYCFR | 1:T015-016 | 2618.2224 | 51.3 | 2617.9827 | 51.3 | 2617.9858 |
| FDTNSHNDDALLKNYGLLYCFRK | 1:T015-017 | 2746.3174 | 48.8 | 2746.0808 | 48.8 | 2746.0784 |
| NYGLLYCFR | 1:T016 | 1147.5485 | 48.7 | 1147.4387 | 48.7 | 1147.4392 |
| NYGLLYCFRK | 1:T016-017 | 1275.6434 | 44.3 | 1275.5251 | 44.3 | 1275.5205 |
| NYGLLYCFRKDMDK | 1:T016-018 | 1764.8328 | 45.5 | 1764.7002 | 45.5 | 1764.6938 |
| KDMDKVETFLR | 1:T017-019 | 1380.7072 | 26 | 1380.5765 | 26 | 1380.571 |
| DMDKVETFLR | 1:T018-019 | 1252.6122 | 29.7 | 1252.4894 | 29.7 | 1252.4861 |
| VETFLR | 1:T019 | 763.4229 | 26 | 763.351 | 26 | 763.3476 |
| VETFLRIVQCRSVEGSCGF | 1:T019-021 | 2129.0398 | 63.2 | 2128.9224 | 63.2 | 2128.9021 |
| IVQCR | 1:T020 | 617.3319 | 12.4 | 617.2723 | 12.4 | 617.2694 |
| SVEGSCGF | 1:T021 | 784.3062 | 21.8 | 784.233 | 21.8 | 784.229 |
