# Supplementary figures and images for: Structural and functional characterization of recombinant human growth hormone isolated from transgenic pig milk
Source: PLoS One. 2020 Jul 31;15(7):e0236788. doi: 10.1371/journal.pone.0236788 (PMC7394428; doi:10.1371/journal.pone.0236788)

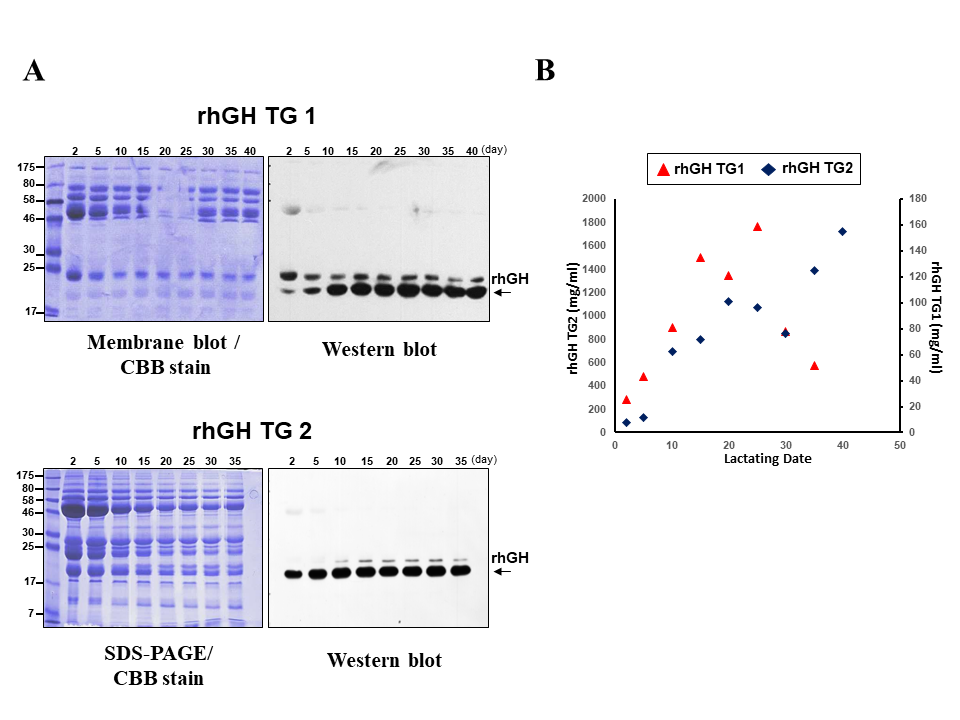

Supplement: S1 Fig — A) Total milk protein separation was assessed by Coomassie brilliant blue (CBB) staining. rhGH protein expression was confirmed by Western blot assay. rhGH TG1: rhGH transgenic pig identification number 39–3 (rhGH-LYY 17, F0 generation). The lactation period was 40 days. Protein samples were loaded onto 12% SDS-PAGE gels after 10X dilution in distilled water. After Western blot assays, the same gel was stained with CBB. Crude milk loading volume: 0.5 μL. Primary antibody treatment 1:3000, secondary antibody treatment 1:5000. rhGH TG2: rhGH transgenic pig identification number 94–2 (hGH-LYY 27, F0 generation). The lactation period was 35 days. Protein samples were loaded onto 13.5% SDS-PAGE gels after 10–20X dilution in distilled water. Crude milk loading volume: SDS-PAGE: 0.5 μL, Western blot: 0.1 μL. Primary antibody treatment 1:5000, secondary antibody treatment 1:10000. (B) Quantification of rhGH protein expression by ELISA assay during lactation period. (TIF) [file pone.0236788.s002.tif]
